# Uma Nova Via para Oclusão Coronariana com Elabela?

**DOI:** 10.36660/abc.20210662

**Published:** 2021-09-01

**Affiliations:** 1 Serviço de Cardiologia do Instituto Orizonti Belo Horizonte MG Brasil Serviço de Cardiologia do Instituto Orizonti, Belo Horizonte, MG - Brasil

**Keywords:** Oclusão Coronária, Angina Microvascular, Miócitos Cardíacos, Circulação Colateral, Biomarcadores (Elabela)

A Oclusão coronariana total (OCT) caracteriza-se por uma obstrução com duração estimada em mais de 3 meses e está presente em cerca de 15-18% de todas as coronariografias realizadas.^1^ As manifestações clínicas podem variar desde quadros assintomáticos, passando por angina estável, até graus variados de disfunção ventricular. Isto depende principalmente do grau da circulação colateral. Sendo pobre, pode levar a comprometimento da viabilidade dos miócitos e associar-se a vários graus de necrose e/ou miocárdio hibernante. Na presença de colaterais bem desenvolvidas (Rentrop grau III), o paciente pode ser assintomático e ter função contrátil preservada.^2^ Portanto, a formação de uma circulação colateral bem desenvolvida, principalmente antes da oclusão do vaso, é fundamental para a preservação dos miócitos.

Apesar dos avanços no tratamento clínico da angina estável, muitos pacientes portadores de OCT são submetidos a procedimentos intervencionistas. Destes, a maioria é encaminhada para cirurgia de revascularização miocárdica ou intervenção coronariana percutânea (ICP) com técnicas mais avançadas, mas também com maior risco de complicações. O estudo de mecanismos potencialmente estimuladores da arteriogênese e angiogênese (formação de circulação colateral) abre oportunidades para o desenvolvimento de medicamentos que, por sua vez, evitariam intervenções desnecessárias e/ou poderiam beneficiar pacientes com sintomas refratários ao tratamento clínico convencional otimizado e que são maus candidatos às intervenções (exemplo: leito distal fino, doença coronariana difusa).

Nesta edição dos ABC, em um estudo transversal, Yavuz e Kaplan^3^ observaram a relação entre os níveis de elabela com OCT e a presença de circulação colateral. Há plausibilidade biológica para suportar esta associação. A estimulação do sistema apelinérgico, seja pela apelina ou elabela sobre os receptores APJ, estimula a proliferação vascular.^4^ Enquanto a apelina promove predominantemente a angiogênese, isto é, vascularização mais capilar, que é importante em regiões peri-lesionais. Já a elabela é responsável pela arteriogênese, promove a formação de novos vasos com diâmetro de até 2 mm de diâmetro,^5^ que é o aspecto mais importante para a formação de circulação colateral.^2^

Foram necessários aproximadamente 30 anos entre a descoberta do receptor APJ até o aparecimento dos primeiros estudos clínicos,^4^ mas serão necessários mais tempo para estudos prospectivos comprovarem a associação entre elabela e formação de novos vasos em adultos. Será que a partir destes estudos deste sistema teremos o desenvolvimento da primeira droga efetiva, que funciona sendo agonista de um sistema para tratamento da doença coronariana crônica? Atualmente as drogas eficazes atuam inibindo a agregação plaquetária, reduzindo o LDL colesterol, estabilizando as placas ateromatosas, reduzindo o consumo miocárdico de oxigênio, mas, não, estimulando a neoformação vascular.^6^ Será interessante a mudança de paradigma, e teremos que aprender a manejar tais medicamentos, cuja lógica pode não ser a de uso por tempo indeterminado.
