# Supplementary material for: Uma Nova Via para Oclusão Coronariana com Elabela?
Source: Arq Bras Cardiol. 2021 Sep 1;117(3):511. [Article in Portuguese] doi: 10.36660/abc.20210662 (PMC8462969; doi:10.36660/abc.20210662)
Supplement: Translation

# Translation: A New Pathway to Coronary Occlusion with Elabela?

**Affiliations:** 1 Belo Horizonte MG Brazil Serviço de Cardiologia do Instituto Orizonti, Belo Horizonte, MG - Brazil

**Keywords:** Coronary Occlusion, Microvascular Angina, Myocytes, Cardiac, Collateral Circulation, Biomarkers (Elabela)

Total coronary occlusion (TCO) is characterized by an obstruction with an estimated duration of more than 3 months and is present in approximately 15–18% of all coronary angiographies performed.^1^ Clinical manifestations can range from asymptomatic, through stable angina, to varying degrees of ventricular dysfunction. This mainly depends on the degree of collateral circulation. If it is poor, it may compromise the viability of myocytes and associate with various degrees of necrosis and/or hibernating myocardium. In the presence of well-developed collaterals (Rentrop grade III), the patient may be asymptomatic and have a preserved contractile function.^2^ Therefore, the formation of a well-developed collateral circulation, especially before vessel occlusion, is essential for the preservation of myocytes.

Despite advances in the clinical treatment of stable angina, many patients with TCO undergo interventional procedures. Of these, most are referred for coronary artery bypass grafting surgery or percutaneous coronary intervention (PCI) with more advanced techniques, but also with a higher risk of complications. The study of mechanisms potentially stimulating arteriogenesis and angiogenesis (collateral circulation formation) opens up opportunities for the development of drugs that, in turn, would avoid unnecessary interventions and/or could benefit patients with symptoms refractory to optimized conventional clinical treatment and who are poor candidates for interventions (e.g., thin distal bed, diffuse coronary disease).

In this issue of ABC, in a cross-sectional study, Yavuz and Kaplan^3^ observed the relationship between elabella levels with TCO and the presence of collateral circulation. There is biological plausibility to support this association. Stimulation of the apelinergic system, whether by apelin or elabella on APJ receptors, stimulates vascular proliferation,^4^ while apelin predominantly promotes angiogenesis, that is, more capillary vascularization, which is important in peri-lesional regions. Elabella is responsible for arteriogenesis, promotes the formation of new vessels with a diameter of up to 2 mm,^5^ which is the most important aspect for the formation of collateral circulation.^2^

It took approximately 30 years from the discovery of the APJ receptor to the first clinical studies,^4^ but more time will be needed for prospective studies to prove the association between elabella and new vessel formation in adults. We may wonder whether based on studies of this system the first effective drug, which works as an agonist of a system for the treatment of chronic coronary disease, will be developed. Currently, effective drugs inhibit platelet aggregation, reducing LDL cholesterol, stabilizing atheromatous plaques, reducing myocardial oxygen consumption, but not by stimulating vascular neoformation.^6^ A paradigm shift will be interesting, and we will have to learn how to handle such medications, whose logic may not be one involving use for an indefinite time.
